# Bio-Based Cellulose Acetate Films Reinforced with Lignin and Glycerol

**DOI:** 10.3390/ijms19041143

**Published:** 2018-04-10

**Authors:** Patricia Gontijo de Melo, Mariana Fornazier Borges, Jéssica Afonso Ferreira, Matheus Vicente Barbosa Silva, Reinaldo Ruggiero

**Affiliations:** 1 Institute of Chemistry, Federal University of Uberlândia, Uberlândia MG 38408-902, Brazil; patriciagontijo@iftm.edu.br (P.G.d.M.); mariana-for@hotmail.com (M.F.B.); mvicente.quim@gmail.com (M.V.B.S.); 2 School of Dentistry, Federal University of Uberlândia, Uberlândia MG 38408-902 Brazil; jessica.afonsof@gmail.com

**Keywords:** membranes, cellulose acetate, lignins, glycerol, macaúba endocarp

## Abstract

Two sets of four cellulose acetate (degree of substitution = 2.2) were incorporated with lignin extracted from the macaúba endocarp, before and after being chemically modified to sodium carboxymethyl-lignin and aluminum carboxymethyl-lignin, respectively. The eight membranes were prepared by the casting method after dissolution in acetone and embedded with lignins (0.1% *w*/*w*), one without modification (CAc-Lig) and two chemically modified (CAc-CMLNa) and (CAc-CMLAl), compared to membranes of pure acetate (CAc). In group II, in the four membranes prepared, glycerol was added (10% *w*/*w*) as a plasticizer. The membranes were characterized by a number of techniques: thermal (differential scanning calorimetry (DSC) and thermogravimetric analysis (TGA)), morphological (scanning electron microscope (SEM) and atomic force microscopy (AFM)), structural (X-ray powder diffraction (XRD)), hydrophobic (contact angle and water vapor permeability), and thermomechanical (dynamic thermal mechanical analysis and tensile tests). The results show that despite some incompatibility with the cellulose acetate, the incorporation of the lignin in a concentration of 0.1% *w*/*w* acts as a reinforcement in the membrane, greatly increasing the tension rupture of the material. The presence of glycerol in a concentration of 10% *w*/*w* also acts as a reinforcement in all membranes, in addition to increasing the tension rupture. In this study, glycerol and acetate both increased the compatibility of the membranes.

## 1. Introduction

Until recently, human activity on the planet spared no effort in the search for products and materials that facilitated our existence and improved the quality of human life. Thus, the degradation of natural resources, namely water, soil, and air, has elevated exponentially the concern for the preservation or at least dramatic reduction of our impact on the environment and therefore the preservation of life on earth. An awareness of these problems has led the general population, governments, regulatory bodies, and the scientific community to seek alternatives that minimize environmental impacts while preserving the maximum quality of life. The important actions used to achieve this aim are numerous, but particularly the emergence of green chemistry, the search for sources of alternative energy (non-fossil fuel), the use of biomass in energy production, and new materials are important research objectives. With these objectives in mind, we constructed membranes or films that can be used in diverse applications, such as packaging (food films), barriers in periodontal reconstitutions [1] and so on. Our main concern was to use materials derived from vegetal, biocompatible, non-toxic, and biodegradable biomass waste. The matrix of the membranes is made of commercial cellulose acetate (CA) with a degree of substitution (DS) of 2.2 and the other components are a lignin extracted from the endocarp of the fruit macaúba (*Acrocomia aculeata*) and glycerol, co-produced by a transesterification reaction of oil in the production of biodiesel. The lignin was used in natura and chemically modified in a very low concentration. All these components caused profound changes in the properties of the membranes.

In recent years, agro-polymer-based materials have attracted significant attention due to their large availability, biocompatibility, renewability and biodegradability [2]. Lignin, the second most abundant polymer of vegetal origin, has many potential applications. Its principal use is as lignosulphonate, a co-product of the sulfite method used for the bleaching of paper pulp, which is soluble in water and is used directly without modification in a wide variety of applications [3].

CA is one of the most important organic esters obtained from natural sources. Its applications include fibers, membranes, films, and plastics [4]. The most important parameter related to the solubility of CA is the DS. CA with a DS of >2.5 is soluble in dichloromethane, with a DS of 2.0–2.5, it is soluble in acetone, dioxane and methyl acetate, while for a DS of >0.8, it is soluble in acetic acid [5,6]. CA can be widely used in the food packaging industry because of its versatility in the processing method, including extrusion, injection, and compression, as well as its comparatively low cost and performance ratio compared to other polymers. CA is also lightweight, microwavable and has good optical properties [7]. In addition, CA biocomposites have good barrier properties, prolonging the shelf life of foods because they protect food against the attack of microorganisms and oxidation [8].

Membranes can be classified as asymmetric or symmetric. This classification is related to the existence or not of a homogeneous structure throughout the membrane thickness [9]. Asymmetric membranes are often produced by phase inversion with partial solvent evaporation and immersion in a non-solvent. These membranes present a superficial film, which is responsible for the separation, and a porous sub-layer, which usually works as a mechanical support. Swelling agents, such as salts and water, can be added to the polymeric solution and act as pore formers [10]. Symmetric membranes can be porous with uniform pore size throughout their thickness, or dense without pores [9,11]. The characteristics, properties and applications of these membranes can be controlled by the preparation methodology. Its applications include reverse osmosis (water desalination), hemodialysis, and the separation of organic mixtures [12,13].

The growth of glycerin in the market has been the subject of discussions among those who see it as a good occurrence and those who refer to it as a “tsunami of glycerin”, an allusion to the devastating effects that it could cause in the economy [14]. Glycerol is a plasticizer widely used due to its good plasticization efficiency, large availability, biocompatibility, and low exudation [15,16].

Lignin is responsible for the mechanical resistance of plants, the protection of tissues from microorganism attack [17], and also has antioxidant properties [18], qualities that are highly appreciated in a raw material used as a reinforcement in mixtures of polymers [19]. The direct use of lignin without any modification can be made, as in the case of lignosulfonate, which is a lignin resulting from pulping. However, due to the complex and amorphous structure of the native macromolecule, its incorporation into polymer matrices requires functionalization. Many examples of functionalization of lignins are described in the literature [20]. Chemical modification is an alternative to changing the behavior, solubility, and compatibility of lignin added to various polymer matrices, and as a result, a reinforcing effect is observed [21]. Simple modifications of lignin, as well as its use in the native form, may provide inexpensive and environmentally-friendly applications of the macromolecule, for example, etherification of lignin and incorporation of different metals in the macromolecule, as alternatives for capturing pollutants from water [22,23].

## 2. Results and Discussion

**Figure 1:**
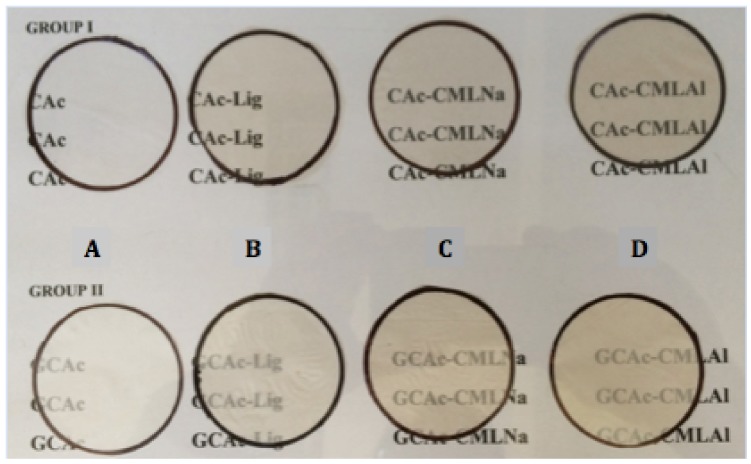
Eight membranes of cellulose acetate (CA) produced. The letters A, B, C and D refer to the membranes of groups I and II described in this figure.

Figure 1 shows the two series of membranes prepared by the method described in the experimental section. 

Group I represents the four membranes without glycerol (CAc, CAc-Lig, CAc-CMLNa and CAc-CMLAl). Group II represents the four membranes embedded with glycerol at 10% *w*/*w* (GCAc, GCAc-Lig, GCAc-CMLNa and GCAc-CMLAl).

**Figure 2:**
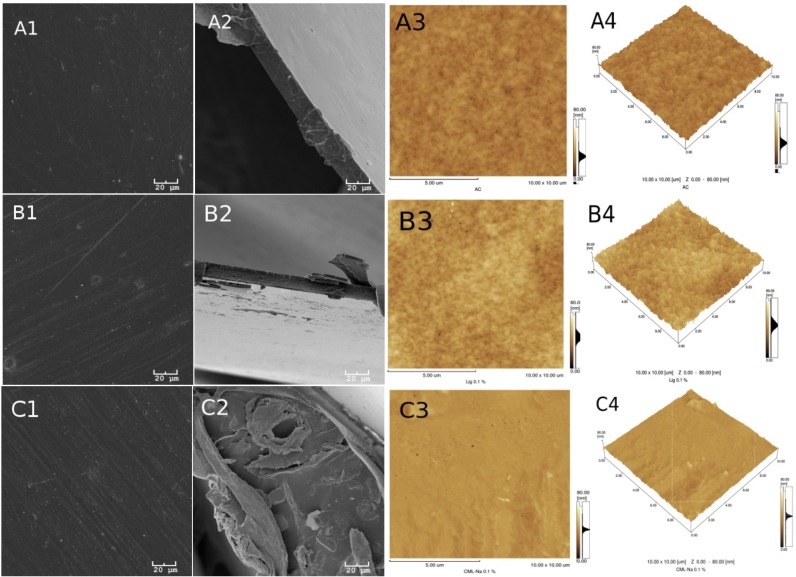

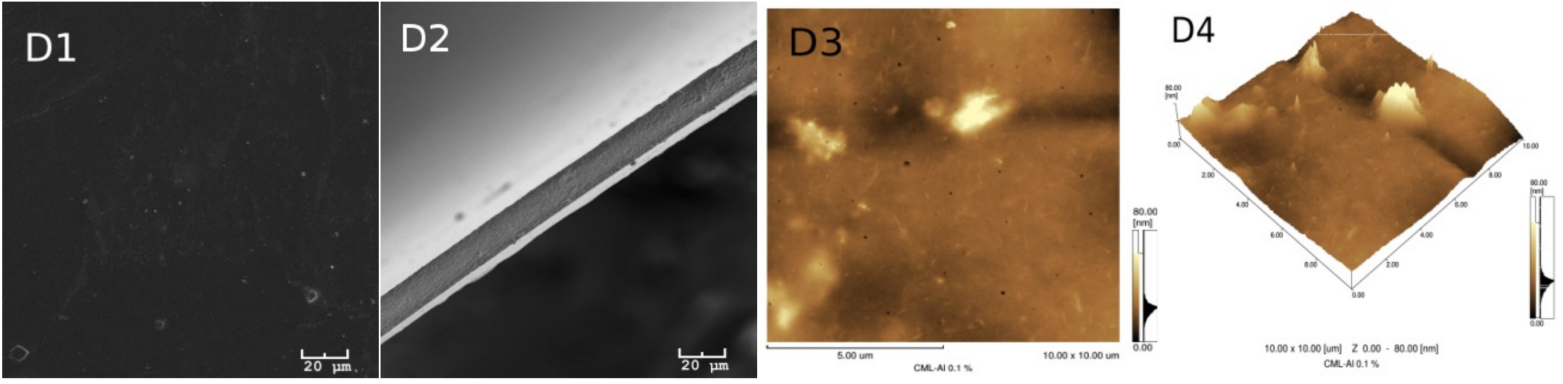
The letters A, B, C and D refer to the membranes of groups I and II described in Figure 1. Numbers 1, 2, 3 and 4, in A1 through D4, refer to surface SEMs (1) and fracture (2) of the membranes and the two-dimensional (3) and three-dimensional (4) AFM of the membranes of group I.

**Figure 3:**
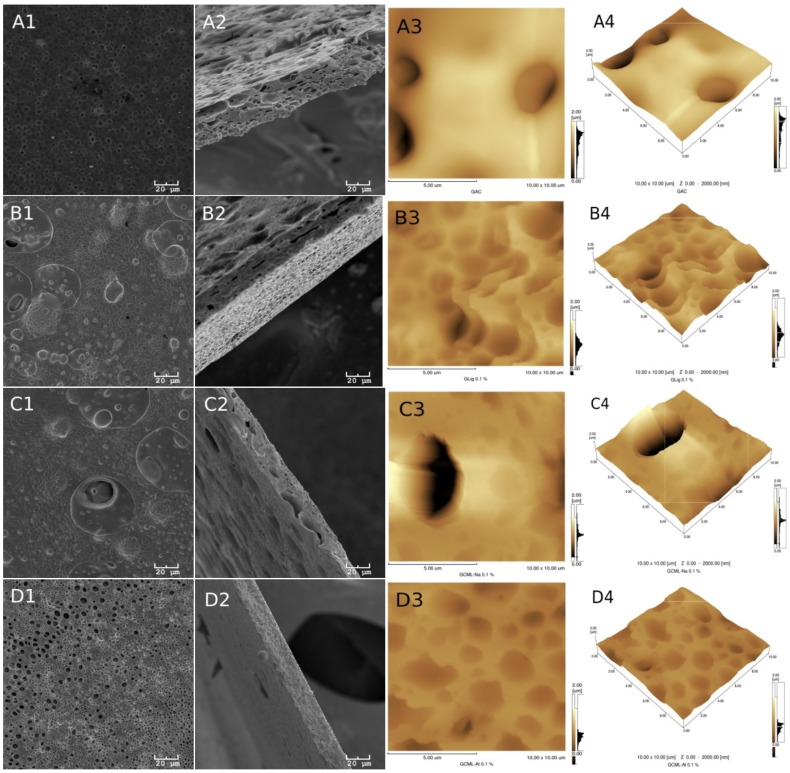
The letters A, B, C and D refer to the membranes of groups I and II described in Figure 1. Numbers 1, 2, 3 and 4, in A1 through D4, refer to surface SEMs (1) and fracture (2) of the membranes and the two-dimensional (3) and three-dimensional (4) AFM of the membranes of group II.

Figure 2 and Figure 3 show the morphologies of the group I and II membranes, respectively. These figures show the scanning electron microscope (SEM) images of the surface in 1 (A1, B1, C1 and D1) and its fractures in 2 (A2, B2, C2 and D2). In addition, the atomic force microscopy (AFM) images are two dimensional in 3 (A3, B3, C3 and D3) and three dimensional in 4 (A4, B4, C4 and D4).

A certain superficial homogeneity and low porosity for the samples of group I are observed in images 1 and 3. However, after incorporating glycerol (group II), a significant presence of pores can be observed in all of the images of Figure 3.

The presence of lignin, however, is more difficult to see in the images, probably due to the low concentration of this material in the samples. Many of the dark spots observed in the AFM and SEM images actually correspond to the presence of deeper pores in the membranes.

Table 1 provides the average roughness data of the membranes for the two groups. In group I, the incorporation of lignin in the CA in the absence of glycerol shows an increase in the average roughness of the membrane with the unmodified lignin and the lignin incorporated with aluminum (Al). For the samples incorporated with glycerol, this effect is reversed, although for all membranes of group II, the roughness increased above 94% in relation to the membranes of group I. In the case of group II, the roughness is associated with the greatest attraction between acetate and glycerol, forming the largest number of pores observed on the membranes. While in the presence of lignin, this interaction is diminished, reducing the formation of deeper pores.

**Figure 4:**
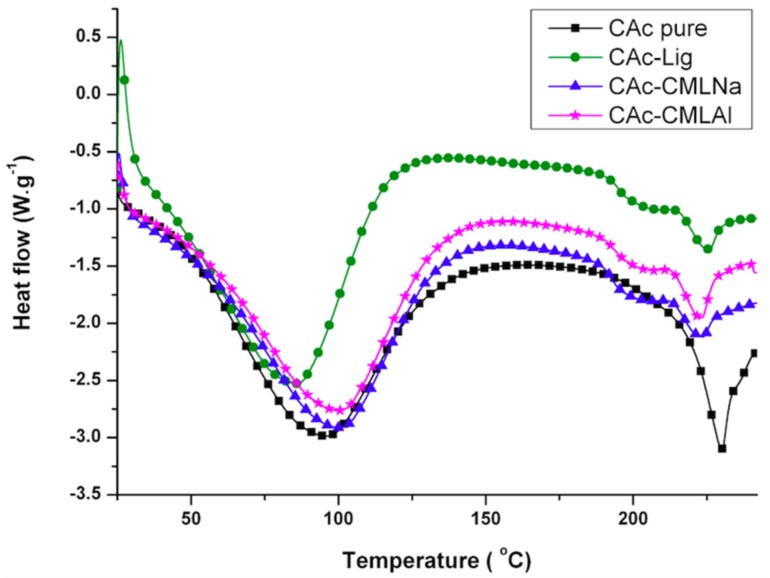
Differential scanning calorimetry (DSC) curves for the membranes of group I.

**Figure 5:**
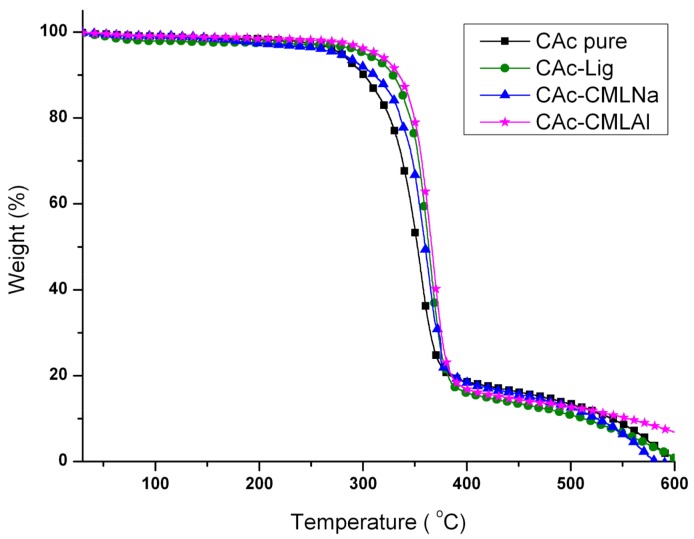
Thermogravimetry (TG) curves for the membranes of group I.

**Figure 6:**
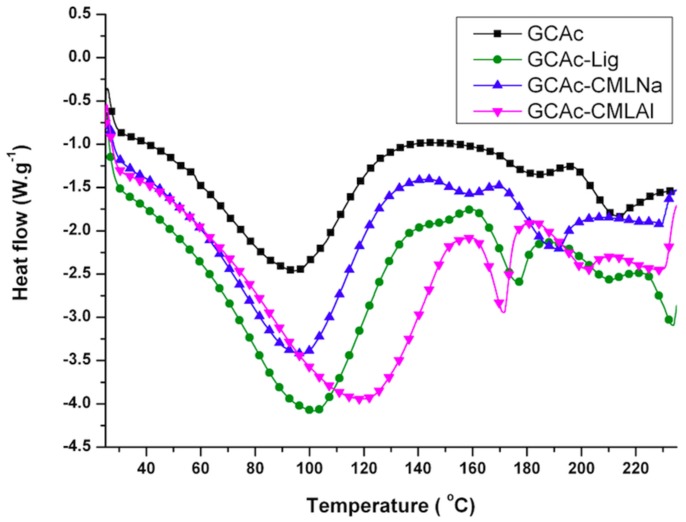
DSC curves for the membranes of group II.

**Figure 7:**
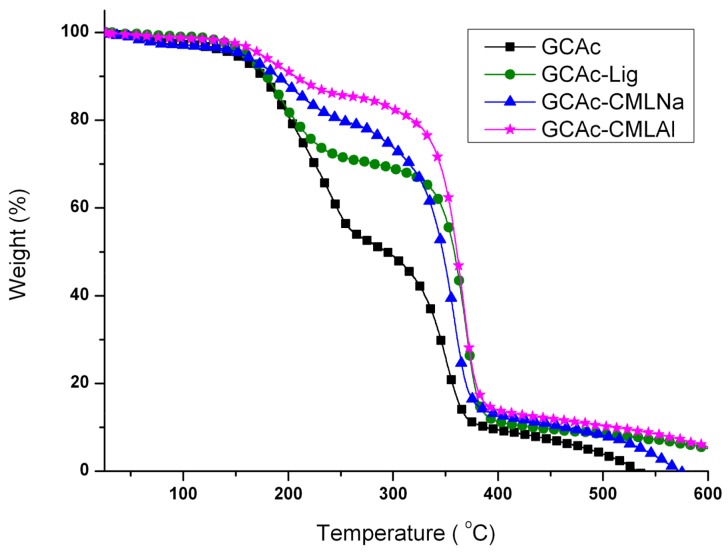
TG curves for the membranes of group II.

The thermal analysis (thermogravimetric analysis (TG) and differential scanning calorimetry (DSC)) shows that when glycerol is added to the membranes (Figure 4, Figure 5, Figure 6 and Figure 7), the mass loss in the samples (TG) of group II is greater and a new endothermic band appears at ~150–160 °C (DSC). By analyzing the DSC curve in Figure 5 (data from group I), it is observed that the pure acetate has an endothermic band at 230 °C. This band, in the presence of lignin, is shifted to 220 °C, with a new endothermic band produced at 200 °C that is wider and of lower intensity. It is likely that the band that changes from 230 to 220 °C is related to the compatibility between the organic part of lignin and the CA, while the new band at 200 °C refers to the mismatch between the inorganic part of the lignin macromolecule and CA. As shown in Figure 6 and Figure 7, with the addition of glycerol in these samples (group II), new bands occur. For CAc with no added lignin, the endothermic band changes from 230 °C (group I) to 215 °C and a new band appears at 180 °C (group II). The decrease of 15 °C for the acetate pure band in the presence of glycerol can be attributed to the compatibility (mixture) between the two components of the membrane, while the smaller and less intense band at 180 °C can be attributed to the “free” glycerol in the sample. This behavior seems to repeat for the CAc-CMLNa sample, where there is a greater shift in the band from 230 °C relative to CA in the sample without glycerol, to 190 °C for the sample with glycerol, which shows the high compatibility between these two components. This behavior is expected since this lignin sodium carboxymethylated (CMLNa) is a water-soluble polyelectrolyte at pH > 4.5 [22] and can therefore interact more favorably with the glycerol incorporated into CA. The other two lignins, native (unmodified) and aluminium carboxymethylated (CMLAl), are insoluble in water and must have a lower compatibility with glycerol. This is evident for the samples with the large endothermic band from CAc at 230 °C in Figure 4, and the endothermic more intense bands from GCAc-Lig and GCAc-CMLAl of glycerol at 175 and 170 °C, in Figure 6. 

Glycerol is added to the membranes at a concentration of 10% *w*/*w*, although not completely associated with the CA, and lignin acts as a reinforcement in the membrane. This result can be seen in Table 2, which provides the onset temperatures (T_Onset_) of degradation of the samples. It can be seen in Table 2 for the membrane of pure cellulose acetate (CAc) that T_Onset_ ranges from 264 to 310 °C after incorporating glycerol (GCAc). In all four samples, the incorporation of glycerol acts as a reinforcement in the membrane, but with different degrees of incorporation. In the case of GCAc, ~46% of the incorporated weight is “free” (glycerol that does not interact significantly with the structure of the membrane) and ~49% of the incorporated weight is linked effectively with the matrix of CA (see weight loss % for the first and second events in Table 2). For the other three samples, where lignins are also inserted, however, the percentage of glycerol incorporation into the matrix is much higher. In the case of GCAc-Lig, only ~31% of the mass of glycerol is “free”, while 68% is effectively interacting as the matrix of CA. In the other two cases, the incorporation is more effective. GCAc-CMLNa and GCAc-CMLAl are only 10% and 13% glycerol “free”, and 90% and 88% grafted into CA, respectively.

Table 3 shows the changes in permeability (flux) and contact angle measured for samples of group II compared with group I. For the membranes of group I, the lowest contact angle is for the CAc-CMLNa sample, while the highest is for lignin in natura. This is a consequence of the greater hydrophilicity of the membrane with the sodium carboxymethyl lignin (CAc-CMLNa) against the higher hydrophobicity of the sample incorporated with not modified lignin (CAc-Lig). The diffusion across membranes, however, despite having similar values when considering the mean deviation, follows the descending order of CAc > CAC-Lig > CAc-CMLNa ≥ CAc-CMLAl.

The incorporation of glycerol in the membranes (group II) causes significant alterations in the permeability of water vapor and contact angle. The increase in permeability is above 90%, largest in CAc (95.7%) and CAc-CMLNa (94.3%), followed by CAc-CMLAl (92.9%) and CAc-Lig (90.7%). The contact angle decreases in this case from 7% to 13%, with the largest drop in the CAc-CMLNa sample (12.9%), due to its greater hydrophilicity, and the smallest in the CAc-CMLAl sample (7.2%).

X-ray diffraction shows an estimate of the crystallinity of the samples. Table 4 provides the crystallinity index provided by the equipment based on calculations using a Lorentz curve. For group I, the crystallinity decreases in the order of CAc > CAc-Lig > CAc-CMLNa > CAc-CMLAl. Lignin is an amorphous macromolecule, unlike cellulose, even in the acetate form [24], which has linear chains. The carboxylation of lignin further reduces its crystallinity by the incorporation of the sodium carboxymethyl group. The exchange of sodium for aluminum should further reduce the crystallinity if we take into account that the largest number of oxidation of Al relative to Na should lead to the emergence of large modified lignin aggregates around the Al [23]. Within group I, the crystallinity loss goes from 15% to 24% relative to the pure acetate membrane, whereas in group II, the loss is smaller, going from 3% to 9%. After the incorporation of glycerol, this sequence is maintained, but with different values. Interestingly, the crystallinity of the pure acetate membrane decreases with the addition of the glycerol, perhaps because of having ~50% of “free” glycerol in the membrane, while for the other three samples, this parameter decreases. Another possibility is the interaction of hydrogen bonding, which must occur between the glycerol and the chains of CA, causing its displacement when the mixture take place due to the incorporation of the glycerol in the acetate matrix of the membrane.

**Figure 8:**
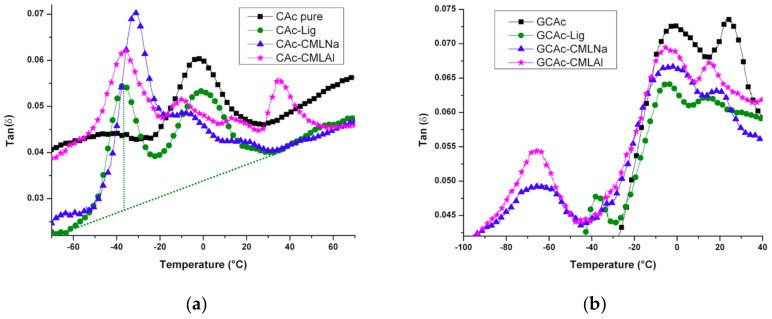
Tan δ versus temperature for the membranes of groups I (**a**) and II (**b**).

The analysis of the membranes by dynamic thermal mechanical analysis (DMTA) shows (Tan delta, Figure 8a,b) profound changes when the glycerol is embedded in the membranes.

For the membranes of group I, the incorporation of lignin causes changes in the thermomechanical events, despite the low concentration of this component in the samples (Figure 8a). For the pure acetate (CAc), the glass transition temperature (T_g_) at −3.5 °C changes its position after incorporating lignin. For the CAc-Lig sample, an increase in the Tg to −2.5 °C occurs, probably due to the greater thermal stability of this component observed in Table 3 from the TG data (T_Onset_ and T_Max_) and in Figure 5. New signals appear at lower temperatures of around −40 °C when lignins are incorporated into membranes. For CAc-Lig, a new signal appears at −37.6 °C with a similar intensity that can be attributed to the amorphous organic structure of the natural lignin that causes a decrease in the crystallinity of the membrane (see Table 5). The carboxymethylation of lignin causes remarkable effects on this T_g_ signal. For the CAc-CMLNa membrane, this signal has the highest intensity and occurs at T_g_ = −32 °C. A higher intensity indicates a greater dispersion capability of the stored energy and the highest T_g_ indicates that the presence of sodium enhances the thermal stability of the sample. For the CAc-CMLAl membrane, the T_g_ of at least four distinct signs appears in the picture. Signs appear at −37, −10.8, 12.8 and 34 °C. The signals at positive temperatures show the reinforcement caused by the presence of lignin in the membrane, probably due to the aluminum in the structure. Alternatively, the T_g_ signal at −37 °C shows a similar effect attributed to the natural lignin in the CAc-Lig membrane, but due to the lower intensity shows less dispersion ability of the stored energy. In Table 3, the T_Onset_ and T_Max_ values confirm this similarity.

**Figure 9:**
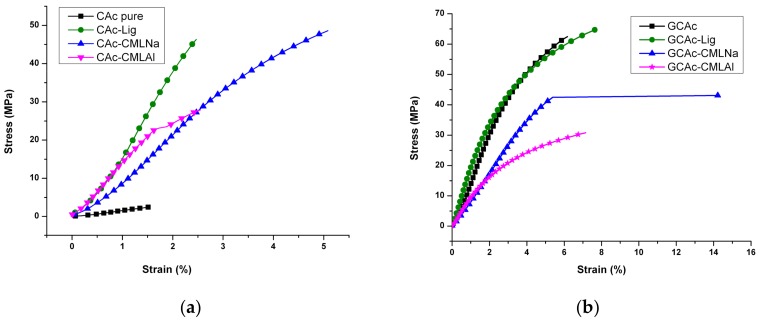
Stress-strain curves for the membranes of groups I (**a**) and II (**b**), respectively.

Tensile tests of the membranes confirm these observations of reinforcement caused by the incorporation of lignins. As seen in Figure 9a, the higher stress/strain occurs in the CAc-Lig membrane with a rupture stress of 46 MPa. The rupture stress of the CAc-CMLNa membrane is also high (48 MPa), but with a greater deformation of 5.1%. This membrane undergoes the greater deformation.

The second membrane with a high rupture stress/strain is CAc-CMLAl, which has a tensile strength of 27 MPa, but with low deformation (2.5%). Compared with CAc pure, all lignins promote reinforcement in the membranes.

After adding glycerol into the membranes (Figure 9b), these mechanical and thermal properties are changed. The results of determination of the T_g_ by measures of tan delta (Figure 8), we note a better compatibility of the added components, due to the presence of glycerol. This can be checked by the proximity of the T_g_ results between −10 and 40 °C for all samples of group II, compared with the samples of group I (see Figure 8a,b). These results show reinforcement in the membranes caused by the presence of 10% glycerol, with positive T_g_ values from 15 to 24 °C. The incompatible portions of the lignins can be identified by T_g_ at very low temperatures: −37 °C for GCAc-Lig, −66 °C for GCAc-CMLNa and −67 °C for GCAc-CMLAl. We can see in these values, the big T_g_ temperature drop for the two lignins modified with metals. In this case, however, the intensity decreases considerably when compared to the sample without glycerol, which shows less capacity to disperse the stored energy.

These effects can be confirmed by tensile testing, as shown in Figure 9. The greatest strengthening occurs in the membrane of GCAc (see Figure 9b) and in the membrane with lignin (GCAc-Lig) incorporated.

Taking as a parameter the stress of rupture of the membranes, we can see from Figure 9 that the presence of glycerol reinforces all membranes. For CAc, the stress/strain relationship at break changes this parameter from 3 Mpa/1.6% to 64 Mpa/6.3%, which is the largest change. In the sequence of the samples, the changes are from 46 Mpa/2.5% to 6 5Mpa/7.8% to CAc-Lig, from 48 Mpa/5.1% to 44 Mpa/14.4% for CAc-CMLNa and from 28 Mpa/2.6% to 31 Mpa/7.2% for CAc-CMLAl. We observe very different behavior for the GCAc-CMLNa membrane after glycerol is embedded, which does not significantly alter its stress of rupture, but nearly triples its deformation capacity. This behavior shows that besides the traction reinforcement, the composite has a synergy among the three components, leading to it acting as a plasticizer on the matrix, which causes a significant stretch before breakage.

## 3. Materials and Methods

### 3.1. Materials

Commercial cellulose acetate (CA) was supplied from Sigma-Aldrich^®^ (St. Louis, MO, USA), with M_w_ = 30,000 g·mol^−1^ and 39.8 wt % of degree of substitution in acetyl content. The glycerol and acetone used were supplied from the Brazilian Chemicals VETEC (São Paulo, Brazil) with 99.5% and 99% purity, respectively. The macaúba endocarp, from which the lignin was extracted, was courtesy of the extractive industry of macaúba oil, from the Paradigma Óleos Vegetais in Patos de Minas, Minas Gerais, Brazil.

### 3.2. Extraction and Modification of Lignin

To each 20 g of endocarp (powdered) of macaúba was added 150 mL of aqueous sodium hydroxide solution of 1.0 mol·L^−1^ under agitation. After 2 h of standing, the mixture was filtered. The filtrate was acidified to pH 2 to precipitate the lignin. The precipitated lignin was filtered and thoroughly washed with distilled water to 80 °C to remove residues of fat and sugars. Then, the lignin was dried in an oven at 50 °C for 24 h [25]. These lignins were chemically modified to sodium carboxymethyl-lignin (CMLNa) and after to aluminum carboxymethyl-lignin (CMLAl) by the literature methods [22,23].

### 3.3. Membrane Preparation

The membranes were prepared from the polymeric matrix of commercial cellulose acetate [25] (10.0% *w*/*v*) using acetone as a solvent and were divided into two groups, as shown in Table 5.

Group I: membranes without glycerol (CAc pure), and incorporated with lignin extracted from the macaúba endocarp (CAc-Lig), CAc-CMLNa and CAc-CMLAl at a concentration of 0.1% *w*/*w*. Group II: membranes with glycerol (10.0% *w*/*w*): GCAc formulated with lignin extracted from the macaúba endocarp, GCAc-Lig, GCAc-CMLNa and GCAc-CMLAl at a concentration of 0.1% *w*/*w*.

After complete dissolution of the CAc in acetone, the lignin and derivatives (CMLNa and CMLAl) were mixed and stirred for 24 h, after which time the membranes were cast onto a glass plate using an adjustable film applicator knife-type with high precision from 0 to 10,000 μm, with an adjustment of 1 to 1 μm, a working width of 15 cm as ASTM D823-53, supplier TKB Erichsen Commercial and Technical Ltd. (São Paulo, Brazil). The formed membranes remained under vacuum at room temperature for 24 h to remove residual solvent and were then stored in a desiccator.

### 3.4. Membrane Characterization

The DS of the material was determined in order to characterize it as cellulose acetate. The DS is the average number of hydroxyl groups that are replaced by acetyl groups in the glycosidic units [5]. The determination of the DS is by the method described in the literature [26].

SEM was performed on a ZEISS instruments model LEO 940 A (Zeiss, Germany), at an accelerating voltage of 10 kV. Prior to analysis, the samples were coated with an ultrathin gold layer in a sputter coating system.

The surface morphology was characterized by AFM using a Shimadzu SPM-9600 (Shimadzu, Japan) in non-contact mode, suitable for soft or fragile surfaces, which might be damaged by the probe, as is the case for the membrane.

X-ray diffraction analysis was performed on a Shimadzu XRD-6000 apparatus (Shimadzu, Japan) with a target of Cu at 40.0 kV and 30.0 mA, with a continuum scan at 2° θ·min^−1^. The crystallinity index (Icr) were obtained by the Lorentz equation provided by the computer program of the equipment that analyzes the diffraction patterns and provides the crystallinity index based on the crystalline region of the sample, expressed in Kcps.Deg.

To determine the water vapor permeation, tests were performed in triplicate using Payne’s cup technique (ASTM D1653-08). By this technique, films with a 30 mm diameter were kept for 24 h in a desiccator. After the films were weighed, the average thickness was measured using a Mitutoyo digital micrometer (0–25 mm, Mitutoyo, Japan) with measures in five different regions of each film. Payne’s cup with distilled water was placed in a desiccator and the mass variation (loss) due the flux through the membrane was measured by weighing the system during the first 12 h at 1 h intervals.

Contact angle measurements were performed on the samples by the literature method [22]. Membranes with a length of 40 mm and a width of 10 mm were fixed on a glass slide and measurements were performed at 25 ± 3 °C with relative humidity of 60 ± 5%. Drops (50 μL) of distilled water (measured in a Hamilton microsyringe, (Hamilton, USA) were placed on the film at three different points. The images were captured 20 s after the drop of water was in contact with the surface, by a digital camera and analyzed by Surftens software, which fitted the drop’s profile to determine the contact angle.

The TGA of the membranes was made on a Shimadzu model TGA-50 (Shimadzu, Japan). The procedure uses ~7 mg of sample heated in aluminum crucibles, up to 600 °C at the heating rate of 10 °C·min^−1^ under nitrogen atmosphere at a flow of 50 cm^3^·min^−1^. DSC was carried out on a TA instruments model DSC Q-20 (TA Instruments, New Castle, DE, USA), using ~7 mg of sample at a heating rate of 10 °C·min^-1^ from 20 to 250 °C at a nitrogen purge rate of 50 cm^3^·min^−1^.

Thermo-mechanical analysis (DMTA) was carried out with a TA instruments model Q800 (TA instruments) equipped with a tension clamp for films. The samples’ dimensions were 16.0 mm of length × 5.0 mm of width × 200 µm of thickness. A minimum of three samples was measured. The membranes were analyzed in the multi-frequency mode, under the following experimental conditions: amplitude of 8 μm, 1 Hz frequency, a static force of 10 mN and a heating rate of 3 °C·min^−1^ from −140 to 200 °C, and setting an automatic tension of 125%.

The tensile tests were performed using the DMTA equipment (model Q 800, TA Instruments), with a claw type used for measuring the film tension. A pre-load from 1 to 18 N was applied, subjected to a force rate of 0.5 N·min^−1^ at a temperature of 25 °C, the assay was completed until the membrane ruptured. The dimensions used for the test piece were the same used as in the DMA analysis. Each test was carried out three times in order to evaluate the reproducibility.

## 4. Conclusions

All membranes incorporated with 0.1% lignin showed high tensile strength and flexibility after molding and significant improvement of all the mechanical and thermal properties when compared to membranes of pure cellulose acetate.

The addition of 10% glycerol (*w*/*w*), although not fully incorporated into the cellulosic matrix, caused an increased reinforcement in all mechanical and thermal properties. The most significant improvement observed in terms of thermal degradation (T_Onset_) and fracture (stress) was in pure acetate, despite its low acetate incorporation into the matrix (~50%). In the samples with lignin, the mechanical and thermal reinforcements on the membranes did not follow this linearity, but the changes were significant from the point of view of an improvement in certain properties of the membranes.

Among the bio-composite membranes, CMLNa is of particular interest, with and without glycerol. It can be used in filtration systems, for example, for the adsorption of heavy metals and/or dyes from effluents, because of the ease of ion exchange of Na^+^ with other metal ions. The presence of lignin in nature, extracted from macaúba endocarp and then modified as CMLAl may have applications as a form of packaging in the food industry.

## Figures and Tables

**Table 1:** Average rugosity of the membranes.

Membranes	Average Rugosity/nm	
	Group I	Group II
CAc	2.85	249.47
CAc-Lig	4.29	146.48
CAc-CMLNa	1.80	217.15
CAc-CMLAl	5.19	96.33

**Table 2:** Temperatures: onset (T_Onset_), at maximum (T_Max_) and rate of weight loss (WL) for the membranes, obtained from TG and DTG curves.

Membranes	Group I			Group II					
				First Event			Second Event		
	T_Onset_ (°C)	T_Max_ (°C)	WL (%)	T_Onset_ (°C)	T_Max_ (°C)	WL (%)	T_Onset_ (°C)	T_Max_ (°C)	WL (%)
CAc	264	352	84	147	225	41	310	340	48
CAc-Lig	296	360	88	151	191	31	322	362	68
CAc-CMLNa	256	354	83	157	200	10	298	352	87
CAc-CMLAl	305	362	85	168	190	13	310	360	86

**Table 3:** Interactions of the membranes with water (flux and contact angle).

Membranes	Group I		Group II	
	P_H_ (Flux) (±SD) 10^5^/cm^2^·s^−1^	Contact Angle (θ) (±SD)	P_H_ (Flux) (±SD) 10^5^/cm^2^·s^−1^	Contact Angle (θ) (±SD)
CAc	2.290 (0.004)	63.03 (1.36)	53.000 (0.002)	57.16 (1.01)
CAc-Lig	1.85 (0.02)	64.36 (2.02)	19.8 (0.4)	58.13 (1.94)
CAc-CMLNa	1.6 (0.2)	62.73 (2.53)	28.2 (0.6)	54.64 (2.21)
CAc-CMLAl	1.57 (0.01)	64.28 (1.77)	22.10 (0.07)	59.62 (1.56)

**Table 4:** Crystallinity index measured by X-ray diffraction (XRD).

Membranes	Crystallinity Index (I_cr_)/Kcps.Deg	
	Group I	Group II
CAc	0.78	0.73
CAc-Lig	0.66	0.71
CAc-CMLNa	0.63	0.69
CAc-CMLAl	0.59	0.66

Kcps: Kilo counts per seconds; Deg: Degree.

**Table 5:** Formulations used for the preparation of the membranes.

Designation	Group I (0% Glycerol)	Group II (10% Glycerol)
A	CAc	GCAc
B	CAc-Lig	GCAc-Lig
C	CAc-CMLNa	GCAc-CMLNa
D	CAc-CMLAl	GCAc-CMLAl
